# Toward Precision Bioconjugation: Chemical Strategies for Site-Selective Cysteine Conjugation

**DOI:** 10.1021/prechem.5c00323

**Published:** 2026-02-04

**Authors:** Katerina Gavriel, Kevin Neumann

**Affiliations:** Institute for Molecules and Materials, Radboud University, Heyendaalseweg 135, 6525 AJ Nijmegen, The Netherlands

**Keywords:** site-selectivity, bioconjugation, click chemistry, cysteine conjugation, bioorthogonal chemistry, peptides and proteins modification, precision bioconjugation, antibody conjugates

## Abstract

The ability to access atomically tailored complex peptides and proteins provides powerful opportunities for dissecting molecular functions and advancing applications in chemical biology, therapeutics, and (bio)­materials science. Robust precision-engineering strategies are essential to construct well-defined protein architectures while preserving native folding and activity. To do so, chemoselective bioconjugation techniques have been developed to modify specific side chains of amino acids. This allowed for the selective introduction of functionalities on predetermined amino acids. However, ultimate control can be achieved only through site-selective modifications that precisely define both the nature of the linkage and the exact position of conjugation on elongated peptide sequences or fully assembled proteins. Cysteine residues are of particular interest, as their highly nucleophilic thiols offer excellent chemoselectivity and typically occur in low abundance in their reduced form. Here, we examine chemoselective transformations targeting cysteine residues that have been further refined to occur exclusively at predefined positions within a peptide or protein, thereby achieving a high degree of site-selectivity. This review focuses exclusively on chemical strategies for cysteine modification, offering guidance for future synthetic developments within the field of precision chemistry. Achieving this level of precision requires advanced chemical strategies that exploit the local environment of the targeted cysteine. One approach involves leveraging neighboring functional groups, for example, engaging the thiol together with the α-amine or carboxylate to enable selective N- or C-terminal modification, respectively. In such designs, the cysteine side chain may contribute through transient interactions, direct incorporation into the covalent linkage, or the stabilization of the desired product. Recently, a promising strategy has attracted increasing attention in which site-selectivity is enabled by temporary interaction with a proximal amine, thus being applicable to differentiate also between internal cysteines. Together, these strategies highlight that site-selective protein modification has evolved into a powerful tool for the rational design and functional control of complex biomolecules, redefining what is achievable in chemical biology, therapeutics, and biomaterials science. We anticipate that increasingly routine or user-friendly approaches such as the programmable TriTEx method will further accelerate the adoption of precision biomolecule conjugates in both research and industrial settings.

## Graphical abstract

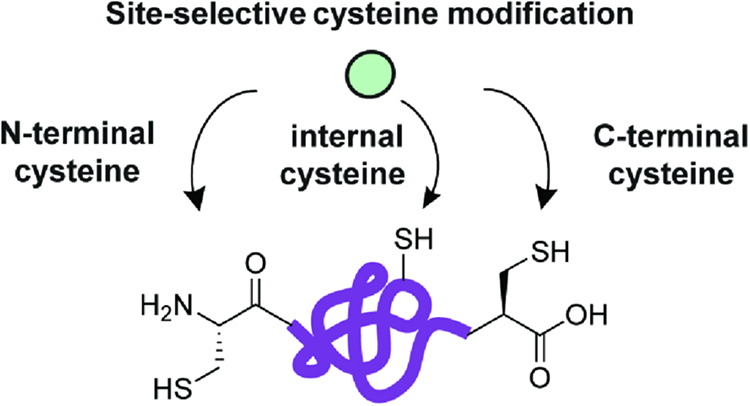

## Introduction

The evolution from broad reactivity toward finely tuned, site-resolved control has opened the field of precision protein engineering. The modification of proteins has revolutionized the study of biological processes and enabled the development of novel therapeutics, biosensing probes, and advanced biomaterials.^1–6^ Proteins possess a large functional diversity, with even minor (off-targeted) chemical modifications having the ability to completely alter the folding of proteins and consequently their function and pharmacokinetic properties.^7^ Therefore, the formation of well-defined molecular architectures and uniform protein samples is essential for their study and application. Chemoselective approaches, specific for chemical functionalities, are widely explored and mostly yield the functionalization of solvent-exposed groups.^8–11^ Thus, chemoselective modifications ensure that a specific type of functional group on the protein is targeted; however, they typically do not discriminate between identical amino acid residues located at different sites within the protein or peptide.

Chemoselective transformations are often focused on targeting cysteine residues because of their low abundance in proteins and their excellent nucleophilicity across various pH values (p*K*_a_ ≈ 8.5).^12,13^ Numerous *in vitro* cysteine-specific bioconjugations have been successfully established for the development of antibody–drug conjugates (ADCs), protein post-translational modification (PTM) mimics, peptide stapling, and living cell imaging.^9,14–20^ Historically, the thiol–maleimide Michael addition has been a widely used cysteine-selective modification. Regardless of the known limitations of the conjugation, that is, the limited stability of the succinimidyl thioether conjugate, the cysteine–maleimide reaction remains a popular modification approach.^21^ Recent efforts focus on improving the stability of the linkage, for example, by self-hydrolyzing maleimides or bifunctional Michael acceptors.^22–27^

Site-selective bioconjugation extends the principle of chemoselectivity by enabling control over not only the reacting functional group but also the precise location of modification, thereby marking a key step toward genuine precision chemistry. Such chemistry allows access to homogeneous protein conjugates with more favorable properties than their heterogeneous counterparts.^4,28^ Additionally, for protein-drug conjugates, the site of ligation and the amount of drug molecules present on the protein are essential knowledge to allow their approval in the pharmaceutical industry. The precise placement of functional groups onto proteins eliminates the potential of detrimental effects on the stability and activity of the said proteins. For example poly­(ethylene glycol) (PEG) is introduced onto proteins to improve their pharmacological properties, however almost all PEGylated proteins present lower activity than their unmodified counterparts because of shielding of the active site by the polymer.^29,30^ Site-selectivity can be achieved by utilizing the excellent selectivity of enzyme recognition toward amino acid sequences, for example, sortase A recognizes the tag sequence LPXTG with excellent precision.^31^ In addition to being highly selective, enzymatic modifications take place under biocompatible conditions, often with excellent yields and high reaction kinetics. On the downside, specific enzymes are often difficult to engineer or are prohibitively expensive, which is why there is considerable interest in purely chemical approaches to bioconjugation.^32^

Here, we reflect on how building on chemoselective conjugation strategies targeting specific thiol side chains gave rise to site-selective protein modification.^33,34^ Historically, especially, the N- and C-termini are sites of interest because they are often solvent-exposed, provide a unique COOH or –NH_2_ in proximity, and for single-chain proteins, there is only one N- and one C-terminus, providing an excellent starting point for achieving site-selective labeling.^35,36^ Therefore, we review cysteine-driven modifications for the late-stage N- and C-terminal tailoring of proteins and peptides (Figure 1). In the strategies presented, it becomes apparent that site-selectivity can be achieved by designing modifications selective for the 1,2-aminothiol or 3-mercaptopropionic acid functionalities, representing the N- or C-terminus, respectively. In addition, we discuss the use of proximity-induced modifications for allowing the selective modification of cysteines within the peptide sequence (Figure 1C). To emphasize precision chemistry in its strictest sense, in this review, we discuss only chemical approaches in which selectivity is not achieved from (precision) biology but obtained from the reaction design.

**Figure 1:**
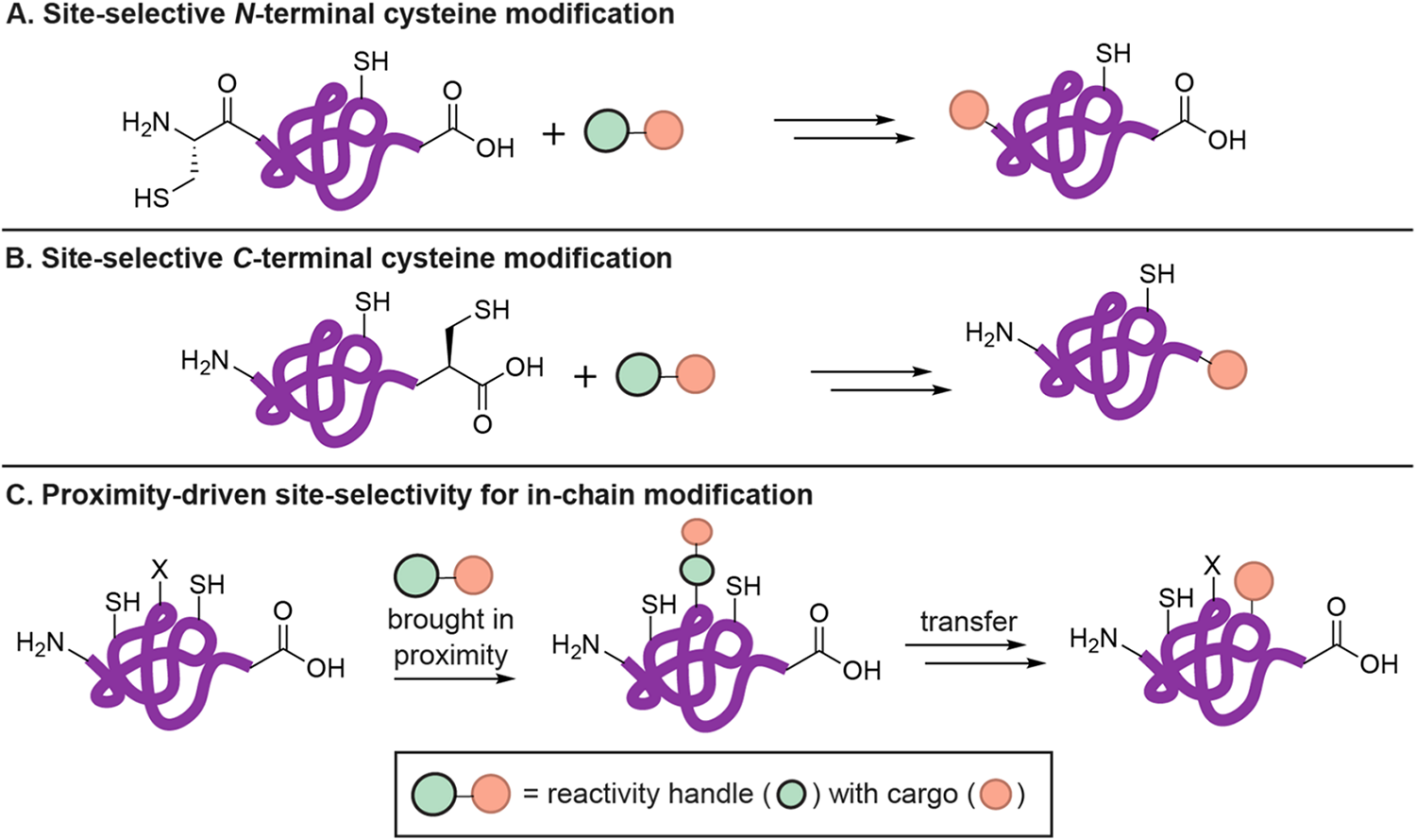
Schematic representation of site-selective (A) N-terminal cysteine modification; (B) C-terminal cysteine modification; and (C) in-chain modification driven by proximity of amino acid side chains in space.

With this article, we intend to establish site-selectivity as a cornerstone of precision chemistry because chemically controlled reactivity at a single defined position enables predictable structure–property relationships in biology and beyond. This precision is crucial when attempting to design programmable conjugation strategies and for translating molecular-level control into functional biomolecular tools.

## N-Terminal Cys Modifications

The N-terminus is an attractive site for modifications as it offers site-selectivity with often minimal disruption of the protein’s folding and natural function. Even though amines are highly abundant in peptides and proteins, the α-amine on the N-terminus is a unique reactive site, as 80% of N-termini are solvent-exposed, as well as a higher basicity than lysines, rendering them reactive at relatively neutral pH.^37,38^ Consequently, N-terminal amino acids are easily accessible and can often be extended without affecting the tertiary protein structure.^36^ Furthermore, the proximity to the side chain results in a new functional group, in the case of cysteine in a 1,2-aminothiol, usually leading to cyclic intermediates and products. Therefore, N-terminal cysteines are often employed for site-selective modification of peptides and proteins due to the unique nature of the α-amine in combination with the potent nucleophilicity of the thiol side chain at a broad spectrum of reaction conditions. While N-terminal cysteines are rare in nature, they are often introduced via genetic engineering, chemical intervention, the use of proteolytic enzymes, or solid-phase peptide synthesis (SPPS).^36^

Arguably, native chemical ligation (NCL) is among the most widely employed N-terminal cysteine modification (Table 1). The reaction proceeds through the chemoselective condensation of a thioester with an N-terminal cysteine-containing peptide (Figure 2A).^39^ In brief, the thiol side chain undergoes a reversible transesterification with the thioester, after which a spontaneous S–N acyl transfer to the N-terminal α-amine forms a mostly irreversible peptide bond.^39,40^ This method was applied for a variety of different purposes, including the ligation of peptide fragments for the chemical synthesis of large proteins, and the modification of proteins and peptides with fluorophores and other molecules.^41–49^ Typically, engineered cysteine residues are readily introduced on the N-terminus by means of SPPS, while mild desulfurization can eliminate undesired thiols on the ligated structure.^50^ Alternatively, after S–N acyl transfer, the liberated thiol may be reacted with maleimide-functionalized cargos to introduce a second functionality via a conjugated addition on the resulting product.^51^

**Figure 2:**
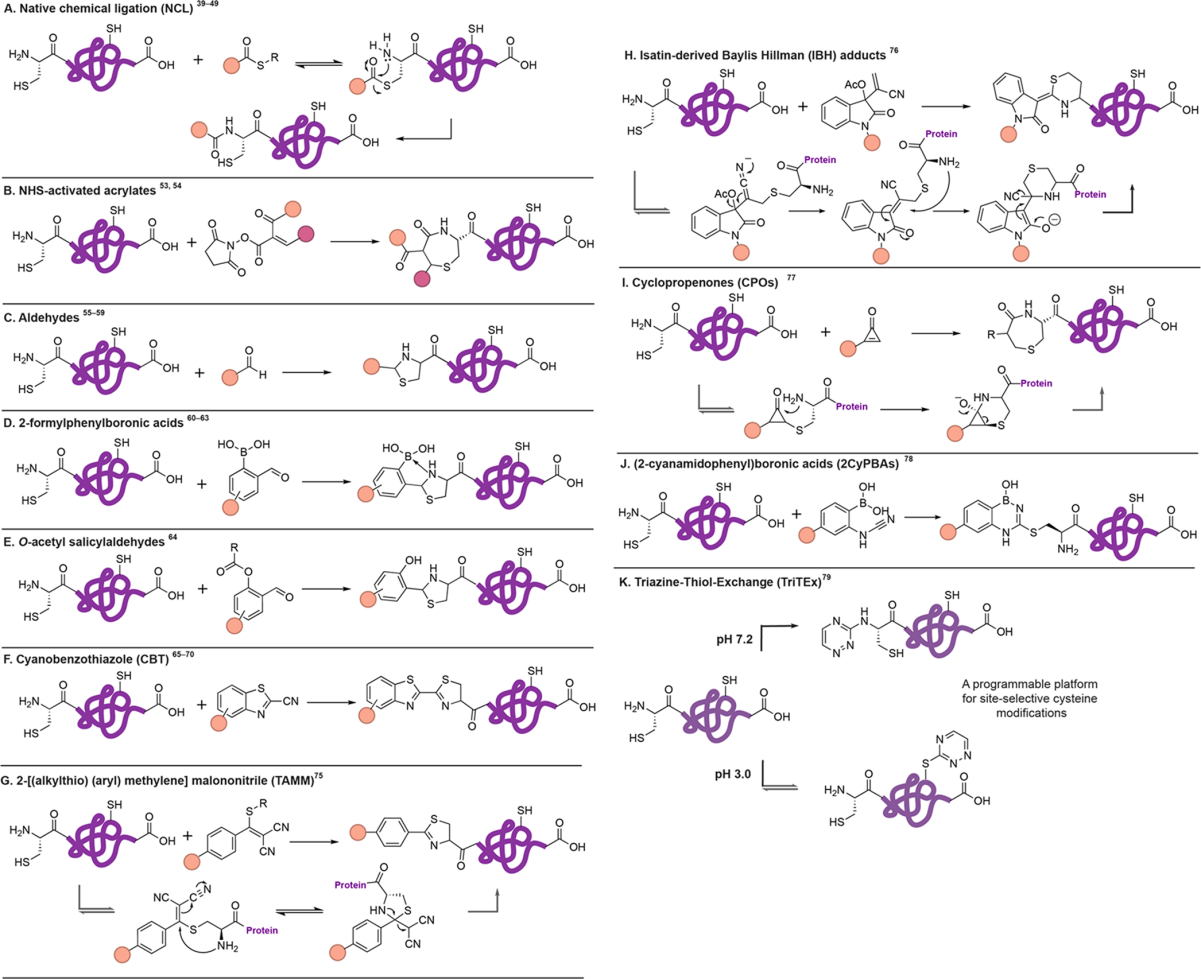
Site-selective N-terminal cysteine modifications. (A) Native chemical ligation (NCL). A transesterification brings the amine close to the thioester, promoting an attack and rearrangement into a peptide bond. (B) NHS-activated acrylates used for N-terminal cysteine modification. Initially, a Michael addition takes place between acrylate and thiol, followed by nucleophilic attack of the α-amine on the NHS. (C) Aldehyde condensation onto 1,2-aminothiol leads to site-selective modification. (D) Condensation of 2-formylphenylboronic acids with N-terminal cysteines. The N–B coordination stabilizes the thiazolidinoboronate product. (E) Stable phenolic thiazolidine formed via the condensation of *O*-acetyl salicylaldehydes with 1,2-aminothiol. Acetic acid is eliminated during the reaction. (F) Condensation between cyanobenzothiazole (CBT) and 1,2-aminothiol resulting in a thioimidate. (G) Reaction between 2-[(alkylthio) (aryl) methylene] malononitrile (TAMM) and 1,2-aminothiol. Thiol exchange followed by attack and rearrangement leads to a stable adduct. (H) Isatin-derived Baylis Hillman (IBH) adducts react with N-terminal cysteines to give bis-heterocyclic species. (I) Nucleophilic attack of the thiol onto the β-carbon of cyclopropenones (CPOs), subsequent intramolecular attack of the α-amine followed by rearrangement forms a functionalized N-terminal cysteine. (J) Reaction of (2-cyanamidophenyl)­boronic acids (2CyPBAs) with N-terminal cysteine leads to site-selective protein modifications. (K) TriTEx enables selective targeting of internal cysteines at acidic pH while preserving N-terminal sites; at neutral pH, it allows for site-selective modification of N-terminal cysteines via an irreversible S,N shift.

NHS esters are widely used in protein labeling and functionalization, they typically react nonsite-selectively with free amino groups.^52^ The group of Gois employed NHS-activated acrylic esters as a two-way means of recognition and functionalization of N-terminal cysteines (Figure 2B).^53^ Silva et al. combined acrylates that can efficiently and rapidly react with cysteines via a Michael addition and NHS esters that react with amines to afford a bifunctional reagent for the rapid stapling of 1,2-aminothiol groups.^24,53^ The group observed no cross-reactivity with other nucleophilic amino acids, concluding that the Michael addition proceeds prior to amidation with the α-amine of the N-terminal cysteine. Additionally, a secondary side chain-to-side chain or side chain-to-tail macrocyclization between lysines and cysteines or C-terminal cysteines was achieved using this methodology, expanding the chemical space of the conjugation.^53^ A year later, the Gois group presented the use of NHS-activated acrylates with dual substitution for the high-yielding multifunctionalization of peptides.^54^ In this case, a peptide was decorated with a fluorescent probe and a poly­(ethylene glycol) chain equipped with an azide, which was further reacted with BCN-containing doxorubicin. Therefore, this strategy allows the incorporation of various payloads onto N-terminal cysteines via a single site-selective and orthogonal bioconjugation handle.

In 1996, Zhang and co-workers introduced a condensation reaction between N-terminal cysteines and aldehydes to form thiazolidines (Figure 2C).^55^ While this method is broadly applicable, it suffers from low reaction kinetics even in large excess of the aldehyde, the requirement of acidic conditions, and the instability of thiazolidine linkages. Since then, much effort is put into improving the kinetics of the transformation even under physiological conditions.^56–59^ To overcome these limitations, the groups of Gois and Gao independently reported the use of 2-formylphenylboronic acids for the site-selective N-terminal modification of cysteines with noteworthy fast kinetics (*k*_2_ ≈ 10^3^ M^–1^ s^–1^) (Figure 2D).^60,61^ Additionally, the authors showed that an *ortho*-boronic acid functionality stabilizes the thiazolidinoboronate product through N–B coordination. Interestingly, thiazolidinoboronate stability was shown to be tunable, meaning that the modification can be reversed.^60–62^ Alternatively, an N-acyl transfer can form stable N-acyl phenolic thiazolidines, constituting an irreversible modification.^63^

Going beyond site-selectivity, achieving programmability, including adjustable stability and on-demand reversibility of reaction pathways, represents a major goal in precision chemistry, enabling spatiotemporal control over molecular architectures. In 2022, the group of Gois showed that *O*-acetyl salicylaldehydes can also be used for the selective modification of N-terminal cysteines with pH-tunable reversibility (Figure 2E).^64^ Interestingly, the stable phenolic thiazolidine did not undergo acetylation, quite in contrast to the earlier reported 2-formyl-3-acetylphenylboronic acid.^63^ This difference led the group to employ DFT calculations and ^1^H NMR analysis in order to understand the underlying mechanism. The authors explained the observed reactivity with a N-terminal amine attack onto the aldehyde, forming a hemiaminal, and subsequent transesterification, which results in acetic acid elimination and iminium formation. Finally, the thiol side chain attacks onto the electrophilic carbon of the iminium forming the final thiazolidine product. Therefore, these results suggest that the presence of the *O*-ester group accelerates phenolic thiazolidine formation. The modification is selective for the N-terminal cysteine such as C-terminal cysteines, internal cysteines, N-terminal lysines, and internal lysines remained unmodified. Finally, the group demonstrated that the phenolic thiazolidines can be selectively hydrolyzed at acidic pH, acting as temporary protecting groups for internal cysteine modification, followed by N-terminal cysteine deprotection and modification with maleimide-presenting functionalities.^64^

Another site-selective conjugation approach was reported by the Rao group. Inspired by the final step in the synthesis of d-luciferin, the authors developed an elegant N-terminal cysteine modification.^65,66^ This methodology involves the nucleophilic attack of the thiol side group onto the electrophilic cyanocarbon of cyanobenzothiazole (CBT) to form a thioimidate (Figure 2F). Subsequent condensation and ammonia release yield the final dihydrothiazole product. This condensation reaction proceeds at physiological conditions with favorable kinetics and stable products, which makes it an attractive fabrication method even for *in vivo* applications.^67–70^ In 2023, Proj et al. synthesized a library of 116 heteroaromatic nitriles containing different electron-donating or electron-withdrawing substituents on the benzene ring and evaluated their stability, reactivity, and selectivity toward 1,2-aminothiol condensations.^71^ 2-cyano benzoxazoles with amines on the 6- or 7-position displayed competitive reaction rates (*k*_2_ = 49 to 188 M^–1^ s^–1^) and excellent selectivity toward N-terminal cysteines. With this work, the group expanded the toolbox of N-terminal cysteine-reacting nitriles with different degrees of site-selectivity and reactivity.

The click reaction between 1,2-aminothiols found in N-terminal cysteines and activated nitriles was further explored, searching for more unique applications. The group of Gao targeted N-terminal cysteines with CBT scaffolds, followed by internal cysteine modification with maleimide-bearing molecules in short peptides and proteins.^72^ This sequential dual labeling of proteins was also utilized after genetically encoding 1,2-aminothiols onto proteins of interest.^73^ In 2019, Huber and co-workers reported the incorporation of 3-(2-cyano-4-pyridyl)­alanines during standard SPPS workflow and the subsequent condensation with N-terminal cysteines to cyclize peptides.^74^ Using this head-to-side chain cyclization approach, an inhibitor for the Zika virus NS2B-NS3 protease was synthesized, presenting high affinity and proteolytic stability.

In 2020, the groups of Tsai and Wu afforded stable 2-aryl-4,5-dihydrothiazole conjugates through reaction between 1,2-aminothiols and 2-[(alkylthio) (aryl) methylene] malononitrile (TAMM) under biocompatible conditions (Figure 2G).^75^ Initially, a thiol-vinyl exchange brings the N-terminus in a preferable orientation for cyclization, enabling elimination of malononitrile to form the stable product. The reaction proceeded with rate constants of 10^1^ M^–1^s^–1^, and the products displayed high resistance toward hydrolysis at acidic and neutral pH for a few days. Using this method, the group fully modified a synthetic peptide but also purified recombinant proteins and proteins in mammalian cells and phages. Similarly, the Kalia group reported the reaction between isatin-derived Baylis Hillman (IBH) adducts and 1,2-aminothiols to form bis-heterocyclic scaffolds (Figure 3B).^76^ The mechanism of the transformation begins with a thiol addition, elimination of the α-acetate group, and eventual intramolecular aza-Michael addition and elimination of the β-cyano group. The reaction proceeds rapidly with rate constants greater than 10^3^ M^–1^s^–1^, enabling quantitative and site-selective protein labeling within minutes at low micromolar concentrations. The modification was applied not only in protein mixtures and in cells but also on 1,2-aminothiols enzymatically introduced at the desired site on a protein of interest.

**Figure 3:**
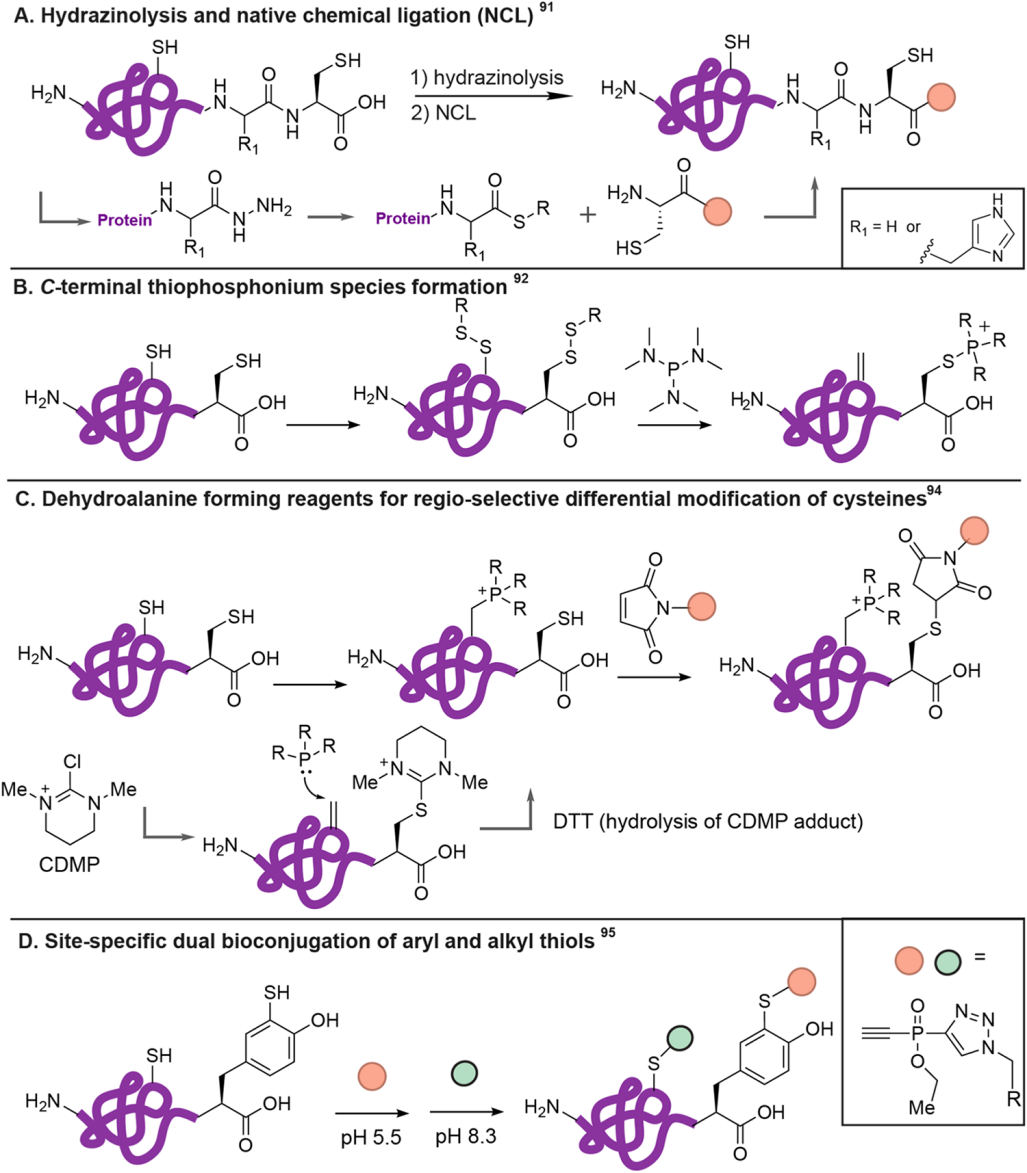
Site-selective C-terminal cysteine modifications. (A) Hydrazinolysis across a His-Cys or Gly-Cys motif followed by *in situ* thioester formation and NCL with a 1,2-aminothiol-containing functionality. (B) Reaction between tris­(dialkylamino)­phosphine reagents and activated disulfide C-terminal cysteines. Formation of activated disulfide prior to the reaction might be necessary. (C) Chemo- and site-selective differential labeling of native cysteines on antibodies using a variety of dehydroalanine reagents (here with CPMP) and subsequent maleimide conjugation. (D) Using aryl thiols on the C-terminus enables orthogonal site-selective dual bioconjugation.

In 2022, the group of Bernardes reported monosubstituted cyclopropenones (CPOs) for the site-selective modification of cysteines residing at the N-terminus of peptides and proteins (Figure 3C).^77a^ CPO is an aromatic three-membered ring with high ring-strain and a large dipole moment, characteristics that contribute to its tendency to undergo nucleophilic additions and ring-opening reactions. The thiol of N-terminal cysteines undergoes a reversible nucleophilic addition onto the least-hindered β-carbon of CPO. Next, intramolecular attack of the amino group onto the CPO carbonyl forms a highly ring-strained bicyclic heterocycle, which rapidly rearranges into a heterocyclic 1,4-thiazepa-5-none bridge. The modification proceeds under mild biocompatible conditions and with high selectivity even in the presence of internal cysteines on the same or on other proteins, as well as the presence of nucleophilic and biological thiols. This method is highly versatile as various functional groups can be added onto CPO via a universal precursor CPO-pentafluorophenol and introduced onto proteins using this methodology.^77a^ The same authors have taken the concept further and have recently reported a CPO-based proximity-driven cyclization linker.^77b^ The cross-linker consists of a flexible linker with a CPO and chloroacetamide residues at its termini. A major advantage of the reported method is the compatibility of CPO chemistry with even large amounts of DTT (in stark contrast to most other cysteine-reactive cross-linkers), resulting in no extra purification step between reduction and cyclization.

All of the strategies described so far involved the formation of a covalent linkage between the N-terminal α-amine and the introduced functionality. This covalent bond is formed either via a temporary covalent linkage with the thiol side chain or the stapling of the 1,2-aminothiol group and therefore the production of a cyclized product. In 2023, the Gois group developed a strategy that directly modifies the thiol side chain of an N-terminal cysteine leaving the N-terminal amine unmodified (Figure 2J).^78^ Reaction of (2-cyanamidophenyl)­boronic acids with N-acetylated cysteine to simulate an internal cysteine resulted in no product; however, use of unprotected cysteine gave high conversions. DFT calculations revealed that the proximal boronic acid orients the N-terminus via the B–N interaction and stabilizes the addition of the cysteine to the cyanamide. Additionally, the free α-amine mediates hydrogen transfer to the thiol at neutral or slightly basic pH and is necessary for the conjugation to proceed. The Gois group proved the site-selectivity by reacting different peptides displaying thiols at the N-terminus or in-chain. The authors observed a single formed adduct with modification only on the N-terminus, while the internal cysteines were subsequently modified using maleimides.^78^

Our group has recently reported an alternative concept to achieve site-selectivity using dynamic covalent chemistry. Building on our expertise on tetrazine-thiol exchange, recently, we reported a programmable approach for site-selective cysteine modification, offering the possibility to target either an N-terminal or internal cysteine (Figure 2K).^79,80^ The strategy relies on a triazine-thiol exchange reaction, a dynamic covalent process whose site-selectivity can be tuned by the pH levels. Under acidic conditions, internal cysteines are selectively modified while preserving N-terminal cysteines, whereas at neutral pH, modification is directed to the N-terminal cysteine. In the latter case, an S–N shift converts the initial dynamic linkage to a stable, irreversible modification. Computational studies support a mechanism in which pH-dependent stabilization of key intermediates governs selectivity, providing a rational framework for the future development of mechanism-guided, site-selective bioconjugation chemistry. While providing excellent selectivity, our approach suffered from low reaction kinetics, making it difficult to adapt to protein modifications; yet, we believe that dynamic covalent reactivity can be further leveraged for precision peptide engineering in the future. Should sulfide-tetrazines be engineered to display similar reactivity, they could serve as versatile handles for conjugation, allowing chemists to install diverse cargos (e.g., dyes, affinity labels, drugs, and polymers). This potential is underpinned by recent methodological advances in the synthesis of structurally complex tetrazines.^81^

**Table 1:** N-Terminal Cysteine Site-Selective Conjugation Approaches are Discussed in This Article

reference	acronym/name	reactivity group	kinetic aspect	reversibility	representative reaction conditions	comment
^39–49^	NCL	thioesters	Often [mM] required with prolonged reaction times	no	depending on thiol additives, conditions can vary but often pH 6.5 to pH 7.4	various peptides, synthesized proteins and recombinantly expressed proteins
^53,54^	NHS-activated acrylates	NHS-activated acrylamides	*k*_2_ = 1.54 × 10^3^ M^–1^ s^–1^ ^53^	no	ammonium acetate, pH 7.0	reagents are bifunctional and allow for stapling
^55–59^	Thiazolidine chemistry	aldehydes	requires long reaction times (up to several days)	yes	acidic pH is often essential (acetate buffer, pH 4.5)	used to generate cleavable linkers for ADCs
^60–63^	Thiazolidine chemistry (FPBA)	2-formyl phenyl boronic acid	*k*_2_ in range of 10^2^ M^–1^ s^–1^ to 10^3^ M^–1^ s^–1^	yes, but acyl transfer affords stable N-acylated thiazolidines	pH values vary topically between 7.0 and 7.4; acyl transfer reagents slightly acidic environment (pH 6.0)	bifunctional maleimides adducts are reported; reaction proceeds at neutral pH
^64^	Thiazolidine chemistry	*O*-salicylaldehyde esters	-	yes	ammonium acetate, pH 7.0 (reversibility at pH 4.5)	allows for controlled reversibility at acidic pH
^65–70^	CBT chemistry	cyanobenzothiazole	*k*_2_ from 10 M^–1^ s^–1^ to 25 M^–1^ s^–1^ ^66,70^	no	PBS, pH 7.4	products may display fluorescence
^75^	TAMM	2-((Alkylthio)(aryl)methylene)malononitrile	*k*_2_ = 4.2 M^–1^ s^–1^	no	phosphate buffer, pH 7.4	used to generate phage-based ADT-cyclic peptide libraries
^76^	baylis Hillman orchestrated protein aminothiol labeling (BHoPAL)	isatin-derived Baylis Hillman adducts	*k*_2_ > 10^3^ M^–1^ s^–1^	no	reaction proceeds under organic or physiological conditions (pH 6 −8)	reaction proceeds in cellular environment
^77^	CPO chemistry	cyclopropenone	*k*_2_ = 3.0 M^–1^ s^–1^	no	NaPi buffer, pH 7.0 with 500 equiv. DTT	CPO are orthogonal with strained alkynes and azides
^78^	benzodiazaborines (BDABs) chemistry	(2-cyanamidophenyl)boronic acids	100 μM, 24 h	no	ammonium acetate buffer, pH 7.0	-
^79^	TriTEx	sulfide-triazine	relatively slow reaction with prolonged reaction time (>12 h)	yes	for N-terminal selectivity pH 3.0; for internal cysteine selectivity pH 7.4	site-selectivity can be programmed by adjusting the pH value

## C-Terminal Cys Modifications

While many strategies have been reported for the enzymatic diversification of the C-terminus of peptides and proteins, not many examples describe the site-selective modification of cysteines residing on that position using a chemical approach.^33,35^ Intriguingly, chemists have introduced 1,2-aminothiol functionalities onto the C-terminus, for example via use of a synthetic dicysteine linker, and reacted said functionality with known conjugations for N-terminal cysteine modifications.^82,83^ Moreover, enzymatic methodologies seem to dominate this area of research while chemical methods remain scarce.^31^ A reason for the lack of chemical tools could be the difficulty in introducing C-terminal cysteines onto peptides during peptide synthesis, as they are prone to epimerization, or piperidine-induced β-elimination and formation of 3-(*N*-piperidinyl)­alanine.^84–86^ Additionally, the 3-mercaptopropionic acid functionality displayed by C-terminal cysteines is more difficult to work with given the challenge of activating and selectively targeting carboxylic groups, as also seen in glutamate and aspartate side chains.^35,87^ Still, as several peptides, proteins, and also the majority of therapeutic antibodies display a native C-terminal cysteine, in contrast to the rare N-terminal cysteines, modification on this site could prove fruitful in proteomics analysis as well as the development of new therapeutics (Table 2).

**Table 2:** C-Terminal Cysteine Site-Selective Conjugation Approaches are Discussed in This Article

reference	acronym/classification	reactivity group	kinetic aspect	reversibility	representative reaction conditions	comment
^91^	hydrazinolysis	hydrazine acetate	Reactions proceed over several days	no	(1) Sodium phosphate, pH 5.8, N_2_H_4_·HOAc (5% w/v), then guanidine·HCl (6 M), 60 °C, 24 h.	allows for diversification at the C-terminus (e.g., via NCL)
					(2) Sodium phosphate, pH 4, NaNO_2_, –10 °C	
^92^	thiophosphonium chemistry	HMPA (reaction with activated disulfides)	At [1 mM] the reaction shows completion after 1 h	Yes, with excess of thiols, see^94^	PB, pH 8.0 buffer	Can be employed for orthogonal Dha formation at internal cysteines
^94^	dehydroalanine forming reagents	2-chloro-1,3-dimethyl-3,4,5,6-tetrahydropyrimidinium (CDMP)	functionalization of Fab is completed after 18 h	yes	(1) TCEP (150 equiv), pH 9.0 BBS EDTA,	Efficiency was demonstrated on antibodies differentiating between C-terminal and internal cysteines
					(2) CDMP, 18 h	
					(3) DTT (500 equiv), 4 h	
^95^	-	Aryl- and alkyl thiols with ethynyl-triazolyl-phosphinate (ETP) as electrophile	For aryl thiols, *k*_2_ = 3.0 to 4.3 M^–1^ s^–1^	no	conjugation to aryl thiols is conducted at pH 5.5; conjugation to aryl thiols is conducted at pH 8.3	Demonstrated to fabricate antibody-drug conjugate carrying two highly cytotoxic cargos, namely MMAE and MMAF

When discussing precision chemistry at C-terminal cysteines, NCL can be regarded as one of the earliest demonstrations. C-terminal hydrazides were proven to be unique reactive groups that can undergo NCL via an *in situ* generation of thioesters.^88^ Although not strictly limited to C-terminal cysteines, hydrazide-containing peptides can be readily accessed through hydrazinolysis of intein fusion precursors, taking advantage of an N,S acyl transfer.^88–90^ In 2013, the group of Macmillan demonstrated that hydrazinolysis across His-Cys and Gly-Cys motifs present on the C-terminus can yield C-terminal hydrazides in the absence of inteins (Figure 3A).^91^ The addition of hydrazinium acetate as a hydrazine source led to the desired hydrazide products. Perhaps more commonly known is the protocol presented by the Liu group, namely, *in situ* thioester generation using sodium nitrite and small thiol molecules, which led to thioesters which were then used for NCL with N-terminal cysteine-containing peptides.^88^ Using this method, ubiquitin G76C was converted into ubiquitin hydrazide, and then thioester formation and NCL with H–CSSK­(biotin)-NH_2_ resulted in C-terminally biotinylated ubiquitin.

A more direct methodology for C-terminal cysteine modification was reported by the group of Chudasama in 2022.^92^ The group described the synthesis of C-terminal thiophosphonium species via reaction between tris­(dialkylamino)­phosphine reagents and disulfide counterparts of C-terminal cysteines (Figure 3B). While typically reactions of disulfides with phosphine reagents result in the reduction of the disulfide bond and release of the free thiols, unsymmetrical alkyl-aryl disulfides (S–SAr) have been reported to allow regioselective phosphine attack on the alkyl sulfur residue, thus forming a thiophosphonium species.^93^ The reactive thiophosphonium species may facilitate the formation of dehydroalanine (Dha). In contrast, Chudasama and co-workers showed that thiophosphonium at the C-terminus is stabilized by the adjacent carboxylic acid. The authors employed the observed stabilization to achieve site-selective labeling of cysteines in model peptides. In the case of internal cysteines, thiophosphonium decomposition toward dehydroalanine was observed, and for N-terminal cysteines, a mixture of thiophosphonium adducts and degradation products was observed. The group demonstrated the feasibility of the modification by installing a thiophosphonium species on the C-terminus of Trastuzumab Fab light chain.^92^

The same team, led by Chudasama, recently reported a strategy that allows differentiation between cysteines in trastuzumab, including light-chain C-terminal cysteines and heavy-chain (HC) hinge-region cysteines (e.g., Cys-Pro).^94^ Site-selectivity is achieved by reaction with dehydroalanine, forming reagents including Mukaiyama reagent (MKYM), EDC, HOTT, and CDMP. While all reagents resulted in dehydroalanine formation of cysteines located in HC, MKYM resulted in minor formation of thioethers, EDC in undesired amide couplings, and HOTT was associated with HC fragmentation side-reactions. In contrast, the use of CDMP overcame these challenges and resulted in clean conversion to the dehydroalanine of internal cysteine in the HC region. Importantly, C-terminal cysteines showed stabilization of the corresponding thiouronium or thiopyridinium species. This feature allowed the authors to conduct a highly efficient sequential modification strategy entailing (i) forming of dehydroalanine at internal cysteines and parallel formation of C-terminal thiouronium or thiopyridinium species, (ii) trapping of the dehydroalanine with phosphines forming a phosphonium, (iii) hydrolysis of the thiouronium or thiopyridinium, and subsequently (iv) labeling with maleimides. Besides studies on the antibody, the authors examined the site-selectivity in somatostatin and observed that C-terminal cysteines experience a stabilization of the thiouronium or thiopyridinium species.

Finally, we refer to the work by de Geus et al., who, although not strictly using native cysteines, reported an elegant approach for labeling C-terminally installed 3-SH-l-Tyr residues.^95^ Importantly, the authors showed that the differences in the p*K*_a_ values allow for the orthogonal reactivity between the aryl and alkyl thiols. After the introduction of the aryl thiol using the enzyme tubulin tyrosine ligase, the aryl thiol was modified at pH 5.5 using ethynyl-triazolyl-phosphinate, and subsequently, the internal cysteine was selectively modified with another ethynyl-triazolyl-phosphinate probe. The authors demonstrated the robustness of their method by the construction of an antibody-drug conjugate carrying two highly cytotoxic cargos, namely, MMAE and MMAF.

## Proximity-Induced Site-Selectivity

In the past two sections, a common theme was the rise in site-selectivity driven by the directly adjacent groups of the two termini. For N-terminal cysteine modifications, we observed that the presence of the thiol in proximity to the α-amine drives the modification on the N-terminus via a temporary covalent linkage or enables the formation of cyclized intermediates or products. For the C-terminal cysteine modification, we noted that site-selectivity can also be the result of stabilization of the product by the presence of the carboxylate group.

In this section, we will discuss how site-selectivity can also be achieved with reactive groups that are much further apart but come together in space in its three-dimensional structure. The concept is closely related to ligand-directed chemistries, a field pioneered by Hamachi and co-workers. These chemistries employ electrophilic warheads such as tosylates, acyl imidazoles, bromo benzoates, sulfonylpyridines, and *N*-acyl-*N*-alkyl sulfonamides.^96–100^ While extremely powerful as bioconjugation tools, even in complex environments, these strategies typically result in nonselective labeling, with the nucleophilic residue not being rationally targeted but rather identified retrospectively. Nevertheless, this chemistry has opened the door to other forms of proximity-directed reactivity, including proximity-induced site-selectivity. Here, we discuss examples in which proximity is induced either by ligands or by linchpin-directed modifications, as well as strategies that employ cysteine residues to direct site-selective modification of adjacent lysine residues, or, conversely, lysine residues to direct cysteine modification (Table 3).

**Table 3:** Site-Selective Conjugation using Proximity Enabled by Cysteine Reactivity

reference	target amino acid	proximity induced via	functionality	traceless	representative reaction conditions	comment
^101^	cysteine	Ligand-directed (ibrutinib analogues)	α-substituted methacrylamides,	yes	Tris buffer, pH 8	recognition element is released via 1,4-elimination
^103^	lysine	cysteine-linchpin	nitroolefin	yes	NaP buffer, pH 7.0	traceless modification is achieved via sequential retro-Michael/retro-Henry reaction
^104^	lysine	Initial cysteine labeling using maleimide-DBCO conjugates	gem-dithioacetate	no	(1) ammonium acetate, pH 7.0	proximity-driven acetylation of histones
					(2) ammonium acetate, pH 8.0	
^106^	cysteine	ligand-directed (peptide) targeting of KELCH domain of KEAP1	iodine-based ethynylbenziodoxolones	yes	Tris buffer, pH 8.0	traceless labeling is achieved by conjugation of the binding peptide toward the aryl residue of the hypervalent iodine species
^107^	lysine	group transfer from proximal	(STEFs)	yes	HEPES, pH 7.5	Fast initial conjugation to cysteine is followed by slower but irreversible transfer to lysine.
^108^	lysine	proximity induced by cysteine labeling	phenolic carbonate	yes (upon reduction of disulfide)	1:1 acetonitrile/PB, pH 7.5	Used to conduct traceless modification of GLP-1 after desulfurization of Cys to Ala
^109^	lysine	proximity induced by cysteine (after disulfide reduction) labeling	pyridazinediones	Yes upon removal of disulfide rebridging reagent	(1) TCEP, PBS, pH 7.4, (EDTA)	Reversible site-selective modification of disulfides using pyridazinediones enables multiple cycles of modifications
					(2) DTT, BBS, pH 8.0 (no EDTA)	
^111^	cysteine	proximity induced by lysine labeling	salicylaldehyde derivatives	Yes (after postfunctionalizing with hydrazide derivatives)	PBS, pH 7.4–7.8	Upon iminium formation with lysine, the aromatic residue experienced a so-called self-activation due to the stronger electron-withdrawing capability, ultimately enabling S_N_Ar reactions with cysteines
^112^	cysteine	recognition sequence (Phe-Cys-Pro-Phe)	perfluoroaromatic reagents	yes	PBS, pH 8.0	-
^113^	cysteine	recognition sequence (Leu-Cys-Tyr-Pro-Trp-Val-Tyr)	aza-dibenzocyclooctyne (DBCO)	yes	PBS, pH 8.0 (6% DMSO)	-
^114^	cysteine	recognition sequence (17mer, referred to as CCMP)	CeCl_3_, styrene derivatives	yes	TRIS, pH 7.4	-

While ligand- and linchpin-based strategies share conceptual similarities with modified group transfer approaches, including the use of proximity to enable site-selective modification, they differ in their mechanistic origin. Linchpin-based strategies rely on a persistent anchoring (at least in the key step) that positions a reactive functionality in the vicinity of the target residue, whereas group transfer strategies typically involve transient modification of a directing residue that subsequently transfers reactivity to a neighboring lysine, frequently (but not always) restoring the original residue in the process. Notably, as many elegant linchpin-based strategies are designed to be traceless in nature, a clear distinction between categories becomes blurred. For this reason, we deliberately refrain from rigidly categorized approaches in this review and instead focus solely on the underlying chemistry that enables site-selective cysteine modification.

Starting with ligand-directed chemistries, we like to refer to the work of London and co-workers who employed high-affinity ligands equipped with a releasable electrophile.^101^ The authors developed a methacrylamide-ibrutinib derivative for site-selective labeling of Cys481 in recombinant Bruton’s tyrosine kinase (2 μM). Elegantly, the authors demonstrated that molecular cargos can be attached to the C-terminal position of the methacrylamide linker, enabling covalent retention of the cargo on the protein even after release of the ibrutinib moiety. While being ligand-directed, the authors’ molecular design enabled the fabrication of a traceless labeling strategy.

In 2020, the group of Rai established a linchpin-directed modification for the single-site labeling of lysine in native proteins.^102^ The group designed a probe where two functional groups connected via a linker of a certain length. One of the functional groups forms imines with solvent-exposed lysines, while the second group covalently and irreversibly labels a lysine in proximity. By precise tailoring of the length and nature of the spacer, lysines at different target sites can be reached. As the number of proteins in a mixture increases, it becomes increasingly challenging to identify a linchpin reagent that enables the precise lysine modification of a single protein. Consequently, the authors hypothesized that a strategy would be valuable in which a linchpin originating from a low-frequency residue is used to modify a high-frequency site, such as directing lysine modification from cysteine.^103^ The authors reported a sequential retro-Michael/retro-Henry reaction scheme that allows rapid chemoselective reaction with cysteine with subsequent irreversible and chemoselective acylation of lysine under mild conditions. The molecular design entailed a nitroolefin for the former reactivity and a tetrafluorophenonol ester for the acylation step. Notably, retro-Henry reactivity involving C–C bond dissociation was achieved by employing a simple pH, rendering the system not only highly efficient and simple to operate but also traceless in nature.

In the same year, the Bernardes group established a three-step protocol for the proximity-driven modification of histones (Figure 4A).^104^ In a first step, histone H3 engineered with a cysteine underwent a Michael addition with a maleimide–dibenzocyclooctyne conjugate, followed by a strain-promoted alkyne–azide cycloaddition resulting in the incorporation of a dithioacetate. This triggered a spontaneous acetylation of an adjacent predefined lysine residue. The group only observed monoacetylated histones, meaning that proximity was essential for the transformation; therefore, more distant lysines were not modified, eliminating the need for purification. Using this approach, the authors tailored histone H3 at the external K9 residue and also the more internal K56 residue. Interestingly, the modification could be potentially reversed using palladium-catalyzed cleavage of the thiosuccimide linkage or thiol exchange via retro-Michael addition. The researchers envision that this method could facilitate the formation of homogeneous samples of acetylated histones and open up opportunities in the study of chromatic structure and function.^104^

**Figure 4:**
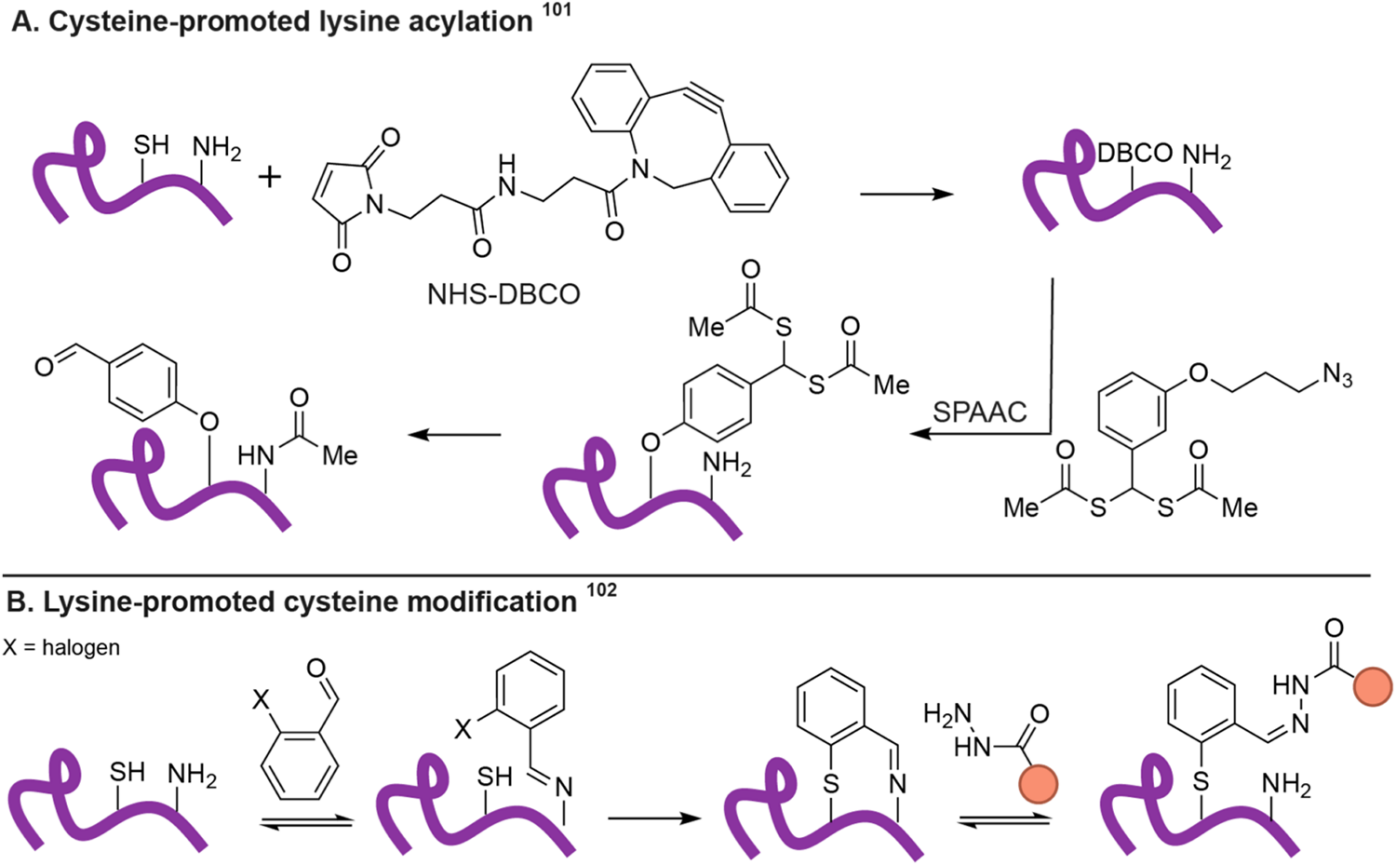
Representative examples of proximity-promoted site-selectivity. For illustrative purposes, we show one example of cysteine-directed lysine modification (A) and, conversely, one example of lysine-directed cysteine modification (B). (A) Cysteine-driven site-selective lysine acylation. A thiol-maleimide reaction followed by strain-promoted alkyne–azide cycloaddition (SPAAC) brings an acylating agent near an adjacent lysine, promoting its acylation. (B) Lysine-promoted site-selective cysteine modification. Upon iminium formation, an aromatic nucleophilic substitution reaction and subsequent dynamic covalent exchange with a hydrazine-bearing functionality of a nearby cysteine is modified.

Especially approaches in which the ligand residue is released and “traceless” conjugation to cysteine residues is achieved are in particular attractive. In this context, the groups of C. Heinis and J. Waser revisited a hypervalent iodine-based ethynylbenziodoxolone (EBX) species, which allows conjugation to cysteines.^105^ By linking a peptide ligand to the arylic residue of EBX, the authors demonstrated that site-selective labeling of Cys434 as part of the KELCH domain of Kelch-like epichlorohydrin-associated protein 1 could be achieved. Notably, the peptide ligand is released upon alkynylation, yielding a site-selective traceless modification.^106^

A clear and powerful example of a group transfer approach was reported in 2019 by the groups of Poulsen and Johannsen disclosing the use of semioxamide vinyl thioethers.^107^ This warhead scaffold was deliberately chosen because of its reactivity toward both thiols and amines as well as its capacity to be conjugated to molecularly complex cargos. The reactivity toward thiols and amines enabled initial fast (and reversible) conjugation to cysteines and subsequent irreversible transfer to proximal lysines. The authors named their probes STEF electrophiles after those scientists who provided the first examples of their preparation, namely, Stachel and Effenberger. When tested on human serum albumin, the STEF electrophile was targeting either of two distinct lysines (K414 and K199), both in shallow hydrophobic grooves, opposed to the commonly targeted K573 and K64, thus underscoring the unique reactivity offered by such transfer strategies. Around the same time, Hymel and Liu employed simple disulfide bonds to bring acylation reagents in close proximity to respective lysine residues.^108^ The authors employed phenolic ester bearing pyridyl-disulfides to form disulfide bonds on human GLP-1. The phenolic ester residue enabled acylation of nearby lysines with carbonate functionalities proven particularly efficient for such transfer. Encouragingly, it was observed that the selectivity was dependent on the location of the installed Cys, thus emphasizing the essential role of proximity. Interestingly, for larger cargos such as C18-diacid-γGlu-bearing phenol, an enhancement in selectivity was observed in comparison to screening with acetate, suggesting that steric bulk further enhances selectivity.

More recently, the groups of Baker and Chudasama reported an elegant approach that enabled labeling of lysine in the form of amide linkages in proximity to disulfide bonds.^109^ This is particularly powerful since cysteines are often present in the form of disulfide bonds, especially in clinically relevant antibody fragments. The authors opted for bromopyridazinedione for disulfide rebridging because of (i) the favorable kinetics and efficiency of the chemoselective cysteine labeling, (ii) the possibility of attaching a transfer acylating reagent in the form of a phenolic ester, and (iii) the ability to remove the pyridazinedione moiety and restore the native disulfide bond.^110^ Optimization of the phenolic ester revealed that ortho-substituted difluoro phenol esters are too reactive, resulting in overacylation, while dichloro- and dibromo ortho-substituted phenol esters presented good stability and selectivity. The high efficiency of this reaction enabled the authors to apply their methodology in multiple cycles of proximity-induced lysine modification, as demonstrated by the dual modification of Ontruzant Fab. In two successive cycles, lysine residues were modified via acylation with an azide-bearing group and subsequently functionalized using 5-FAM-PEG_3_-BCN in cycle 1 and BP-Fluor568-DBCO in cycle 2, ultimately yielding a dually labeled Fab conjugate.

In 2025, the group of Lehn established a protocol for the lysine-assisted arylation of cysteines in proximity (Figure 4B).^111^ The group utilized salicylaldehyde derivatives that can undergo the formation of an iminium with lysines. This covalent dynamic chemistry activated the electrophilic character of the adjacent C–F bond because of the greater electron-withdrawing capabilities of the –CH=N^+^H– motif compared to the parent aldehyde. Therefore, iminium formation self-activated the salicylaldehyde molecule toward aromatic nucleophilic substitution (S_N_Ar) reaction with the nucleophilic thiol side chain of the neighboring cysteine. The group demonstrated the modification of two human antibodies, IgG1 and IgG4, using an activated fluoro-bearing salicylaldehyde derivative with high bioconjugation yields. Cysteines not adjacent to lysines were labeled with a maleimide probe. Next, postfunctionalization of the modified antibodies was performed via a dynamic covalent exchange with a hydrazine-bearing probe. This test confirmed that only samples that reacted with salicylaldehyde derivatives that can undergo both imine formation and S_N_Ar were susceptible to postmodification, while samples reacted with salicylaldehydes that could not form imines were fully functionalized with the maleimide probe instead. Meaning that both reactions are essential for successful and site-selective cysteine modification.^111^

An approach that has not yet been discussed is the use of a peptide recognition sequence to enforce interactions with the conjugation tool itself, thereby bringing it into the proximity of a displayed cysteine residue. A notable example includes the π-clamp reported by Pentelute and co-workers in 2015, which comprises the 4mer H–FCPF–OH.^112^ The cysteine within this short peptide tag displayed significantly enhanced reactivity toward perfluoroaromatic reagents with rate constants of *k*_2_ = 0.73 M^–1^ s^–1^. Interestingly, when single amino acids were substituted, the reactivity decreased (to *k*_2_ = 0.05 M^–1^ s^–1^), showing that each amino acid within this sequence is essential. The authors use the π-clamp to site-selectively modify trastuzumab with monomethyl auristatin F. In addition to this strategy, the group of Pentelute reported a aza-dibenzocyclooctyne-tags (DBCO-tags), describing a cysteine-containing peptide sequence that selectively reacts with DBCO probes, forming a stable linkage.^113^ Similar to the π-clamp, the DBCO-tag enabled site-selective modification of the antibody trastuzumab despite the presence of multiple endogenous cysteines.

In a more recent study, Song and co-workers employed a 17-amino acid cerium-binding sequence for achieving site-selective cysteine labeling.^114^ Notably, cerium enables an oxidative thiol–ene coupling between thiols and vinylic residues within an aqueous environment. The authors showed that only cysteine residues optimally located near the metal coordination site undergo labeling. Ultimately, the authors demonstrated the efficiency of the labeling approach successfully using a recognition sequence-tagged ubiquitin.

These studies highlight how the rational design of recognition sequences can enforce spatial proximity and thereby enable site-selective modification.

## Conclusion and Perspective

While chemoselective tools give rise to biomolecules with well-defined functionalities, site-selectivity allows control of the precise location of the modification. Cysteine plays a critical role in both approaches as its unique reactivity and low natural abundance enable mild and selective protein engineering even in complex biological media. Selective tailoring of the N-terminus can be achieved by reactions recognizing the characteristic 1,2-aminothiol functionality found on N-terminal cysteines. Native chemical ligation is selective for the N-terminus because of a temporary covalent linkage formed via transthioesterification between the thioester and thiol side chain. Other bioconjugation tools, such as NHS-activated acrylates, aldehydes, 2-formylphenylboronic acids, *O*-ester salicylaldehydes, cyanobenzothiazole, and cyclopropenones, react with the 1,2-aminothiol to give a characteristic cyclic product. In contrast, (2-cyanamidophenyl)­boronic acids establish a modification directly on the cysteine side chain, thus preserving the α-amine. In this case, site-selectivity is observed because a B–N interaction between N-terminus and boronic acid preorientates the thiol and cyanamide for a nucleophilic attack to proceed. These solutions offer a diverse set of tools for the site-selective introduction of various functionalities to N-terminal cysteines.

Conversely, C-terminal cysteines constitute a more challenging motif to target, primarily due to the inherently lower reactivity of the carboxylate compared to the N-terminal amine. Still, a couple of selective C-terminal cysteine modifications have been established. Initially, C-terminal hydrazides can be introduced by selectively cleaving C-terminal Aa-His-Cys-OH and Aa-Gly-Cys-OH motifs using hydrazine sources capable of intercepting transient thioesters.^91^ Next, *in situ* generation of thioesters and subsequent NCL allows selective modification of the C-terminus. Interestingly, upon hydrazinolysis, the cysteine residue is removed from the C-terminus. Importantly, this could disrupt the protein sequence which could affect the three-dimensional structure and therefore function of the protein of interest.^115^ However, if NCL follows upon the hydrazinolysis, the original protein sequence is restored, and the protein structure might remain intact. The second methodology involves the introduction of a thiophosphonium species on the C-terminal cysteine side chain.^92^ While the former methodology is not restricted by the functionality introduced via eventual NCL, the latter strategy is restricted to the available tris­(dialkylamino)­phosphine reagents. Still, the field of site-selective C-terminal cysteine modifications is newly established, and further research could potentially lead to more modular and applicable approaches. One attractive direction the authors foresee is the selective trapping of transient C-terminal cysteines and investigating their role using a chemical biology approach.^116^ This review discussed approaches in which, after N-terminal cysteine modification, for example, with CBT, internal cysteines can be targeted with a new functionalization containing maleimide or another chemoselective handle. While this strategy traps the N-terminus, Silva et al. showed that a reversible N-terminal cysteine modification can be used as a temporary protecting group and then selectively removed after internal cysteine modification.^64^ This two-step strategy leads to site-selective modification of internal cysteines in the presence of N-terminal cysteines. Our group has introduced an alternative strategy to distinguish between these two cysteine sites by employing a programmable modification approach based on sulfide-triazines. This method leverages covalent dynamic chemistry, enabling site-selective modification of N-terminal cysteines at neutral pH, while permitting internal cysteine modification under more acidic conditions.

Methodologies to selectively target specific internal cysteines involve proximity-induced reactivity. These reports introduce a new concept by leveraging adjacent functionalities to control the site-selectivity of protein modifications; however, this time by targeting a specific binding site within the three-dimensional structure of the protein of interest. That being said, knowledge about the exact folding of the protein and behavior in certain media is necessary to establish fitting labeling reagents. Additionally, an investigation into the parameters that affect the efficiency of such a system would be necessary to establish a more broadly applicable approach and avoid the reinvention of a reagent for every POI. Beyond proximity-induced site-selectivity in peptide modification, bringing reacting groups in a confined space was used to ligate peptides, label proteins and peptides, transform proteins into biomarker-detectors, and even perform small molecule synthesis.^117–121^

Site-selective bioconjugation is no longer a technical nuance; instead, the authors conclude that it is a defining principle. The examples discussed in this article show that site-specific reactivity elevates biomolecular modification to programmable molecular precision. One can say that site-selectivity is not simply a tool of precision chemistry but in the context of bioconjugation, it is a cornerstone of precision chemistry.

## Vocabulary Section

Site-selectivity: refers to the preferential transformation of one reactive functionality over others of the same type within the same molecule. Achieving site-selectivity is essential for the controlled modification of complex molecules, particularly in late-stage functionalization and chemical biology applications.
Proximity-induced reactivity: refers to reaction acceleration or activation that arises from the enforced spatial proximity of two reactive partners. In bioconjugation chemistry, this can be achieved by taking advantage of the defined folding of proteins, bringing specific residues in close proximity.
Click chemistry: refers to a class of highly efficient, modular reactions that proceed rapidly and chemoselectively under mild conditions to form stable covalent linkages. In the context of chemical biology and bioconjugation, click reactions are often considering water compatible.
Antibody conjugates: are antibodies that have been chemically modified with functional payloads such as drugs, fluorophores, or imaging agents. Site-selective conjugation enables precise control over the number and location of these modifications, yielding homogeneous conjugates with well-defined structure, reproducible properties, and improved biological and clinical performance.
1,2-aminothiol motif: structurally resembles the N-terminal functionality of cysteine residues. Owing to this similarity, it exhibits characteristic reactivity in bioconjugation chemistry, including native chemical ligation and related chemoselective transformations discussed in this article, and is therefore widely exploited for site-selective modification of peptides and proteins.
